# Human-relevant potency threshold (HRPT) for ERα agonism

**DOI:** 10.1007/s00204-018-2186-z

**Published:** 2018-04-09

**Authors:** Christopher J. Borgert, John C. Matthews, Stephen P. Baker

**Affiliations:** 1 Applied Pharmacology and Toxicology, Inc. and CEHT, Univ. FL College of Vet. Med., Gainesville, FL USA; 2 University of Mississippi School of Pharmacy, University, MS USA; 3 Department of Pharmacology and Therapeutics, University of Florida College of Medicine, Gainesville, FL USA

**Keywords:** Endocrine disruptor/disruption, Hazard identification, Potency, HRPT, European Commission, Estrogen system

## Abstract

The European Commission has recently proposed draft criteria for the identification of endocrine disrupting chemicals (EDCs) that pose a significant hazard to humans or the environment. Identifying and characterizing toxic hazards based on the manner by which adverse effects are produced rather than on the nature of those adverse effects departs from traditional practice and requires a proper interpretation of the evidence regarding the chemical’s ability to produce physiological effect(s) via a specific mode of action (MoA). The ability of any chemical to produce a physiological effect depends on its pharmacokinetics and the potency by which it acts via the various MoAs that can lead to the particular effect. A chemical’s potency for a specific MoA—its mechanistic potency—is determined by two properties: (1) its affinity for the functional components that comprise the MoA, i.e., its specific receptors, enzymes, transporters, transcriptional elements, etc., and (2) its ability to alter the functional state of those components (activity). Using the agonist MoA via estrogen receptor alpha, we illustrate an empirical method for determining a human-relevant potency threshold (HRPT), defined as the minimum level of mechanistic potency necessary for a chemical to be able to act via a particular MoA in humans. One important use for an HRPT is to distinguish between chemicals that may be capable of, versus those likely to be incapable of, producing adverse effects in humans via the specified MoA. The method involves comparing chemicals that have different ERα agonist potencies with the ability of those chemicals to produce ERα-mediated agonist responses in human clinical trials. Based on this approach, we propose an HRPT for ERα agonism of 1E-04 relative to the potency of the endogenous estrogenic hormone 17β-estradiol or the pharmaceutical estrogen, 17α-ethinylestradiol. This approach provides a practical way to address Hazard Identification according to the draft criteria for identification of EDCs recently proposed by the European Commission.

**Electronic supplementary material:**

The online version of this article (10.1007/s00204-018-2186-z) contains supplementary material, which is available to authorized users.

## Introduction

The identification of toxic hazards has historically been based on qualitative descriptions of a chemical’s causal role in producing particular types of adverse effects, such as irritation, sensitization, immunotoxicity, nephrotoxicity, hepatotoxicity, reproductive toxicity, teratogenicity, carcinogenicity, genotoxicity and mutagenicity. Only carcinogenic hazards have been further differentiated mechanistically, with the intent to determine whether the cancer risk assessment should assume a threshold or a non-threshold dose–response function. Even that mechanistic distinction, however, is based upon whether the chemical is presumed to first cause another type of adverse effect, either genotoxicity or mutation. Indeed, the word hazard denotes adversity. Accordingly, hazard identification was developed as an early step in the risk assessment process that focuses on adverse effects.

In 1998, the US EPA proposed to evaluate a new category of toxic hazards defined entirely by a general mode of action (MoA), endocrine disruption (EDSTAC 1998). Hazard identification based on MoA presented a new challenge for regulatory agencies because it requires assessing two causal relationships rather than just one (producing an adverse effect specifically by an endocrine MoA). Meeting this new challenge has required the development and validation of new laboratory methods for detecting a chemical’s ability to interact with and modify the functional state of various components of the endocrine system as well as new strategies for using those assays to screen and test chemicals (EDSTAC 1998; ECETOC 2009; OECD 2012). In the US, chemical regulation of the so-called endocrine disrupting chemicals (EDCs) is risk-based, consistent with the manner by which the agency regulates all toxic hazards.

In contrast, the European Union has signaled the intent to regulate EDCs based primarily on hazard. Accordingly, the European Commission (EC) recently proposed criteria for the identification of EDCs (EC 2016). The EC’s proposal, known as Option 2b, is based on the WHO/IPCS definition of an EDC (IPCS 2000). To be identified as an EDC, the proposed criteria specify that (1) it causes an adverse effect, (2) it has an endocrine MoA, and (3) there is a causal link between the adverse effect and the endocrine MoA.

In early 2016, the German Federal Institute for Risk Assessment (BfR) convened a workshop of scientists who held divergent views regarding EC’s proposed criteria for identifying EDCs (Solecki et al. 2017). The consensus statement that emerged supported risk-based regulation of EDCs and noted the importance of using internationally agreed upon protocols for chemical testing. These important agreements have been overshadowed, however, by debate over a more controversial statement whether hazard identification for EDCs should include a consideration of the potency (strength) by which a chemical produces an adverse effect (criterion 1)—i.e., its “toxic potency”. Potency was stated to be important for later steps in risk-based regulation, but not for hazard identification because toxic potency is affected by many factors beyond the scope of hazard identification. Irrespective of that debate, the BfR consensus statement (Solecki et al. 2017) offered little clarity regarding requirements for the third criterion, which, as indicated above, is the important new challenge presented by hazard identification of EDCs. Herein, we propose a way forward for meeting the new challenge of evaluating whether there is a biologically plausible causal link between a chemical’s ability to interact with and modify the functional state of the endocrine system, i.e., its ability to act via an endocrine MoA, and the production of adverse effects.

To establish the biological plausibility of that link, some key properties of the chemical must be evaluated; (1) the affinity of the chemical for, and (2) its ability to modify the functional state (activity) of components of the endocrine system that confer the specificity of hormonal response, i.e., its receptors, enzymes, transporters, and regulatory elements, etc. Only chemicals that exhibit sufficient affinity for and activity with those components can directly modify endocrine signals, and thereby produce a physiologically relevant effect.^1^ Affinity is a measure of the mutual attractive forces that cause two substances to come together and form a semi-stable complex. The concentration of the substance in the vicinity of the biological molecule with which it will interact is a critically important component of the ability of the two substances to interact with one another. Activity is a measure of the ability of the chemical to modify the functional state of the biological molecule with which it interacts, e.g., to serve as an agonist, partial agonist, inverse agonist, antagonist, inhibitor, activator, etc.

Affinity and activity are the determinants of what has been called pharmacological potency in the study of drug mechanisms, but could be more descriptively and generally referred to as “mechanistic potency”. Mechanistic potency is a somewhat different concept than biological potency (Jeyakumar et al. 2011), regardless of whether the biological potency is considered toxic or therapeutic (the potencies by which a chemical produces therapeutic or adverse physiological effects); toxic potency is the concept addressed by the BfR consensus statement. Unlike mechanistic potencies, biological potencies are influenced by pharmacokinetic processes, and it is not necessary to know the MoA for their determination. To avoid confusing the issues addressed by the BfR consensus with those discussed here, we will refer to the specific terms “affinity” and “activity” wherever possible, and will use the term “mechanistic potency” when the inclusive term is appropriate. In the context we use the term here, mechanistic potency relates solely to a chemical’s mode and mechanism of action (e.g., as an agonist of an endocrine receptor subtype), not to the production of therapeutic or adverse effects.

Several characteristics of affinity and activity render these parameters uniquely relevant for the identification of EDCs. First, they are well defined and do not depend on the adverse effect at issue. Instead, they depend only on the ability of the chemical to interact with and modify the functional state of endogenous components of the endocrine system. Second, affinity and activity do not vary with the route of exposure or the toxicokinetic behavior of a chemical (e.g., absorption, distribution, metabolism, excretion), as does toxic potency. Third, they can be measured experimentally for exogenous chemicals and can be compared to the mechanistic potencies of endogenous components of the endocrine system. Fourth, the level of mechanistic potency necessary to convey or interfere with endocrine signals can be estimated empirically and theoretically by investigating these properties for molecules whose effects are causally linked to a particular endocrine MoA versus those that are not.

The evaluation of mechanistic potency is so central to determining whether chemicals can produce effects via an endocrine MoA that it is used within the pharmaceutical industry early in the drug discovery process to identify chemicals that might be useful as drugs to treat disorders of specific endocrine pathways. Similarly, the US EPA’s EDSP-21 program evaluates mechanistic potency in in vitro assays, even before the Hazard Identification step, to prioritize chemicals for further endocrine screening, and the Agency uses mechanistic potency comparisons across different MoAs in its weight of evidence evaluations to identify potential endocrine hazards. A similar type of comparison based on mechanistic potency was suggested for deriving a human-relevant potency threshold (HRPT) to identify chemicals capable of contributing to adverse effects in humans through an anti-androgenic MoA (Borgert et al. 2012). The HRPT concept can be applied to any species with a functioning endocrine system, and can be used to establish or reject the biological plausibility of a causal relationship between a chemical’s hypothesized endocrine MoA and its adverse effects, as required by EC’s proposed criteria for identifying EDCs.

## Overview of HRPT methodology

HRPTs can be expressed as differences in mechanistic potency relative to well-characterized endogenous effectors or human pharmaceuticals. They are derived from mechanistically based in vitro and in vivo assays supported by empirical observations from clinical studies, or in rare cases, epidemiological evidence. To develop an HRPT, data are sought for effectors with high, intermediate, and low mechanistic potencies via the particular MoA, e.g., ERα agonism. In practice, these respective categories will include (1) well-characterized endogenous effectors and/or human pharmaceuticals that produce a predictable physiological effect specific to the MoA; (2) effectors with measurable, but weak mechanistic potencies that produce no physiological effect via the specific MoA; and (3) effectors with mechanistic potencies intermediate to categories 1 and 2 that produce physiological effects via the specific MoA only under conditions of high dose, and often only in combination with a sensitive physiological or developmental state. In addition to data for different effectors of widely varied mechanistic potency, HRPT derivation requires mechanistic potency data from a common endpoint specific to the MoA of interest. The most useful endpoints are those that measure a functional change (e.g., transcriptional activation; cellular proliferation or hypertrophy) rather than merely a molecular interaction (e.g., binding affinity), but deriving an HRPT may require correlating mechanistic potency data from the molecular and physiological levels. This is particularly important when the MoA of interest includes numerous specific molecular interactions.

To address the EC’s requirement, here we illustrate the method for deriving an HRPT by applying the concept to the estrogen MoA. For brevity, we will limit our illustration to comparisons of mechanistic potency via ERα agonists with the understanding that an HRPT derivation for the complete estrogenic system may also require comparisons of mechanistic potencies via each component of the estrogen system. As such, the HRPT proposed here is applicable only to the ERα agonist MoA. Work is ongoing within our group to develop a more comprehensive HRPT proposal for estrogen MoAs.

Several features make the estrogenic system an excellent prototype to illustrate the derivation of an HRPT. The complexity of the estrogenic system makes it one of the most challenging systems for which an HRPT might be developed, yet, the wealth of available data makes it possible to address each intricacy. To modify the estrogen system, an effector must bind to and alter the functional state of at least one of the following; (1) an estrogen receptor, (2) the system for synthesis, activation, desensitization, or breakdown of estrogen receptors, (3) an enzyme or enzymes responsible for synthesis or breakdown, or elimination of estrogens and their precursors, (4) circulating proteins that bind estrogens, (5) the synthesis, activation or destruction of these enzymes and proteins (items 2–4), (6) endocrine and other systems that modify the estrogen system (e.g., LH, FSH, GnRH, inhibin), (7) co-activators, co-repressors, co-integrators, or chaperones, (8) any system that influences the estrogen system through cross-talk, or (9) the presence and availability of estrogen receptor effector systems. These numerous complexities support the proposition that physiological effects may not be predictable across agents that can interact with the estrogenic system and that their characterization may need to proceed on a chemical by chemical basis. Screening of multiple effector systems and with different in vivo physiological models may be required (Germain et al. 2006). Since the primary determinants of mechanistic potency—affinity and activity—underlie all physiological responses produced through hormonal pathways, whether therapeutic or adverse, this implies that deriving HRPTs may require assessments of mechanistic potency for several effectors.

Although the complexity of estrogenic pathways is challenging, the basic principles of receptor, enzyme, and transport kinetics are applicable to each molecular interaction that composes the system. These principles form the conceptual basis for mechanistic potency thresholds that must be exceeded for a chemical to produce physiological effects (Borgert et al. 2013), a concept corroborated through modeling of molecular signaling networks (Zhang et al. 2014). To our knowledge, no theories have been proposed that would replace these fundamental tenets of endocrine pharmacology and toxicology, and thus, it would seem reasonable that HRPTs can be estimated if sufficient data are available. Wealth of data is available for many pharmaceuticals that target estrogenic pathways, e.g., those that are used for hormone replacement therapy, birth control, treatment of estrogen-dependent cancers, and other estrogen system modifications. Furthermore, widespread human exposure to botanical estrogens in the diet provides considerable human data on molecules that are much less potent than active pharmaceuticals, but which nonetheless may produce physiological responses under certain conditions. Finally, extensive characterization of the estrogen receptor system has produced abundant mechanistic potency data for these endogenous, pharmaceutical, and botanical estrogens, as well as for endogenous and exogenous molecules that exhibit weak interactions with components of the estrogen system but produce no discernible physiological effects. Thus, sufficient data are available for molecules spanning a wide range of estrogenic mechanistic potencies and physiological effects to allow estimation of an HRPT for most estrogenic MoAs.

## The estrogen system

The estrogen system is important for the growth, development, and regulation of the female reproductive system and in pregnancy. The estrogenic system is also important for the growth and development of the male reproductive system. It also has important effects on bone, the cardiovascular system, the brain, liver, and other tissues in both genders.

The primary source of endogenous estrogens in the non-pregnant female is the ovarian follicle and its derived tissue the corpus luteum. The three primary endogenous estrogens from this source are 17β-estradiol, estrone, and estriol. The placenta produces these estrogens during pregnancy. Estetrol is an endogenous estrogen produced from estriol and 17β-estradiol by the fetal liver during pregnancy. Chemicals that have some estrogenic activity are also produced in breasts, adrenal glands, adipose tissue, and liver. In males, follicle stimulating hormone (FSH) induces the synthesis of aromatase in the testis, and it induces this enzyme in the adrenal glands and other tissues in both genders. Aromatase converts testosterone and other androgens into estrogens. During menopause, estrone, produced in adipose tissue, is the primary estrogen. Other non-ovarian, non-pregnancy-related endogenous compounds that have some estrogenic activities include 2-hydroxyestrone, 4-hydroxyestrone, and 16-hydroxyestrone, which are metabolites of estrone, metabolites of dehydroepiandrosterone (DHEA) 7-oxo-DHEA, 7α-hydroxy-DHEA, and 16α-hydroxy-DHEA, 7β-hydroxyepiandrosterone, Δ4-androstenedione, Δ5-androstenediol, 3β-androstanediol, 3α-androstanediol, and 5α-androstane-3β,17β-diol produced from testosterone, and 27-hydroxycholesterol produced from cholesterol.

There are numerous sources of exogenous chemicals with estrogenic activities. Plants consumed as food contain a variety of phytoestrogens. Other chemicals that have estrogenic activity include pesticides such as o,p-DDT and chlordecone, surfactants such as octyl and nonyl phenol, phthalates used as UV stabilizers in plastics, polychlorinated biphenyls, once widely deployed as dielectric and coolant fluids in electrical apparatuses and other uses, drug products such as diethylstilbestrol and tamoxifen, and an extensive variety of others.

Most estrogenic chemicals produce their physiological effects via one or more of at least three estrogen receptor subtypes—nuclear receptors ERα and ERβ and the G-protein-coupled membrane receptor GPER30. The nuclear receptors can bind to estrogen response elements to either stimulate or repress gene transcription. They can also tether to other response element binding proteins to act as co-activators or co-repressors of gene transcription. The nuclear receptors can also be transported to the cell membrane where they form complexes with other membrane receptors, e.g., mGluR1, to activate the transduction systems operated by those receptors (Sinchak and Wagner 2012). Some estrogenic chemicals that act as agonists at some of the receptor subtypes under some conditions can act as antagonists at others (Ring and Dowsett 2004; Tzukerman et al. 1994; Harrington et al. 2003). When agonists bind to ERα or ERβ, helix 12 of the receptor protein relocates and seals the agonist binding site (Brzozowski et al. 1997). The bound receptor dimerizes, is phosphorylated on two serine residues, and may then complex with its target system in the cell to produce its physiological effect. The relocation of helix 12 also promotes the association of co-activator with the receptor. Phosphorylations are mediated by several second messenger protein kinase systems including those that are activated through stimulation of growth factor receptors (cross-talk). Some ER antagonists go through the same steps with the exception that helix 12 relocation to seal the binding site is prevented. This also prevents association of the complex with co-activators.

GPER30 is activated by estrogens resulting in Gβγ stimulation of a tyrosine kinase, which phosphorylates and activates SrC-1, which phosphorylates and activates ERK1 and ERK 2 protein kinases (Filardo et al. 2009). In addition to activation of ERK1,2, GPER30 activation can result in a variety of other intracellular signaling actions including formation of IP3 with resultant release of calcium from the endoplasmic reticulum, and formation of cAMP with resultant activation of protein kinase A (Filardo et al. 2009, 2002).

## Control of the estrogen system

### Infancy and childhood

When estrogen levels are low (≤ 6 × 10^−11^ M, as 17β-estradiol) the hypothalamus produces pulsatile gonadotropin releasing hormone (GnRH) release every 1–3 h. GnRH acts at the anterior pituitary to stimulate the release of luteinizing hormone (LH) and follicle stimulating hormone (FSH). GnRH receptors in the anterior pituitary are subject to rapid desensitization. Therefore, only pulsatile release of GnRH will allow for continued pulsatile synthesis and release of FSH and LH. LH and FSH act at the gonads to stimulate the synthesis and release of testosterone (males) and estrogens (males and females). Secretion of FSH and LH is high at birth, and remains relatively high for the first 6 months of postnatal life, but declines to very low levels until puberty. Circulating levels of testosterone and estrogens are also very low during childhood (< 1 × 10^−11^ M, as 17 β-estradiol) (Forest 1989; Klein et al. 1994). Estrogen performs several non-gender specific actions. These include maintenance of vascular endothelium, aortic smooth muscle, some brain tissues, and promotion of the growth, development and maintenance of bone. The estrogens needed by these non-gonadal tissues during childhood are produced by the actions of the enzyme aromatase from adrenal cortical androgens and used in a paracrine or intracrine role in these tissues. Estrogens from these sources do not contribute significantly to circulating estrogen levels (Simpson et al. 2000).

### Puberty

During the last 3 years before the onset of puberty, hormonal changes occur in the hypothalamus that reduce the inhibition of GnRH neurons and allow increasing pulse amplitudes of GnRH to be released. This increasing GnRH release increases the release of LH and FSH leading to puberty onset. In girls, LH and FSH stimulate the production of estrogen from the ovaries, which stimulates the development of female secondary sexual characteristics and the onset of the menstrual cycle. In boys, FSH acts on Sertoli cells in the testis to stimulate testosterone production. Testosterone and other androgens in combination with class 1 cytokines and glucocorticoids stimulate Leydig cells in the testis to express aromatase (Simpson et al. 2000; Bourguiba et al. 2003). In addition to their non-gender specific actions, estrogens are important in male sexual development, spermatogenesis, and development of male sexual behavior. Rising (moderate) estrogen levels (8–9 × 10^−11^ M) act at the arcuate nucleus in the hypothalamus, which sends signals to the preoptic area of the hypothalamus to inhibit GnRH release (Smith et al. 2005). This decreases FSH and LH secretion, which in turn, decreases the production of gonadal estrogen.

### Menstrual cycle

The age at which girls develop menarche in the US is 12.5 years, with fewer than 10% beginning before age 11 and 90% beginning by age 14 (Chumlea et al. 2003). These generally continue until about age 50 (Su and Freeman 2009). The period is measured from day 1 of bleeding (shedding of the uterus lining), until the next bleeding starts (Carr 1998). When fertilization has not occurred, estrogen levels begin to drop near the end of the cycle (Roseff et al. 1989).

### Follicular phase days 1–14 of the cycle

The reduction of estrogen-dependent maintenance support of the uterine lining tissue initiates its breakdown. Falling levels of estrogen (low estrogen levels) near the end of the cycle release the inhibition of pulsatile release of GnRH, resulting in increased secretion of FSH and LH. FSH recruits 5–7 tertiary follicles in the ovaries and stimulates the growth and activity of granulosa cells in the recruited follicles. LH stimulates the growth and activity of thecal cells in the maturing follicles. Thecal cells produce androgens, which are converted to estrogen by aromatase in the granulosa cells. Granulosa cells also express anti-Mullerian hormone, which decreases follicular sensitivity to FSH, which prevents further recruitment of follicles (Durlinger et al. 2002). One or two of the recruited follicles become dominant and these continue to develop and increase their production of estrogen. Increasing estrogen (moderate levels) inhibits the release of GnRH, resulting in reduced levels of FSH and LH. Reduction in FSH and LH causes the non-dominant recruited follicles to fail (die) while the dominant follicles are stimulated by estrogen to continue to develop and to excrete higher levels of estrogen.

### Ovulation

When estrogen levels rise rapidly and remain high (~ 2 × 10^−10^ M) for at least 36 h, estrogen acts at the anteroventral periventricular nucleus in the preoptic area of the hypothalamus, which sends signals to the GnRH neurons, also in the preoptic area, to trigger increased GnRH secretion leading to a surge of LH secretion and a somewhat smaller surge of FSH secretion (Smith et al. 2006). This initiates oocyte meiosis, luteinization of the granulosa and thecal cells, and ovulation (release of the oocyte from the ovary).

### Luteal phase

After oocyte release, the remainder of the follicle becomes the corpus luteum, which produces moderate levels of estrogen and high levels of progesterone. These corpus luteum hormones stimulate endometrial proliferation and endometrial secretion of a variety of cytokines and other paracrine factors (Marchini et al. 1991; Boomsma et al. 2009). The endometrium is in its implantation window 6–10 days post ovulation. Fertilization should occur from 5 days prior to 2 days after ovulation. If fertilization does not occur, then 8–9 days after ovulation the corpus luteum, due to the drop in LH (due to inhibition of GnRH secretion by moderate estrogen levels), decreases in activity and degenerates. The decline of progesterone and estrogen levels causes the endometrium to break down and shed. Fertilization results in an embryo. The outer layer of the embryo, the blastocyst (which becomes the placenta) secretes chorionic gonadotrophin (similar in action to LH), which maintains the corpus luteum, and its hormones, which support pregnancy.

### Menopause

The primary sources of estrogens in post-menopausal women are conversion of adrenal cortical androstenedione and its precursors to estrone in tissues, including adipose, vascular endothelium, aortic smooth muscle, skin, bone, breast, and brain (Simpson et al. 2000). The estrogens produced in these tissues act primarily at or very near their sites of production, and do not contribute significantly to circulating estrogen levels.

### Estrogen in males

In post-pubescent males, the primary source of estrogen is conversion of testosterone to 17β-estradiol by the actions of aromatase. This occurs in the testis and in various non-gonadal tissues including adipose, vascular endothelium, aortic smooth muscle, skin, bone, and brain (Simpson et al. 2000). Since circulating testosterone levels in men are much higher than the level of circulating androgens in women, non-gonadal production of estrogens is much higher in men, and the incidence of estrogen deficiency diseases, such as osteoporosis, is much less prevalent in men than in post-menopausal women (Simpson et al. 2000).

## Development of the HRPT for the ERα agonist MoA

To clearly and concisely illustrate the process for developing an HRPT, we constrain our analysis to the ERα agonist-mediated MoA. Since ERα agonism is the putative MoA for many alleged EDCs, the HRPT for this MoA is immediately useful for hazard identification and its derivation provides a prototype for the development of HRPTs generally.

### ERα agonist prototypes

Several effectors are archetype ERα agonists with high mechanistic potencies producing physiologically relevant responses and clinically measurable estrogenic effects: the endogenous estrogenic hormones 17β-estradiol, estrone and estriol; and the pharmaceutical estrogens ethinyl estradiol and diethylstilbestrol. These define the supra-threshold potency range and serve as reference chemicals for evaluating relative mechanistic potency for an ERα agonist. In contrast, low-potency botanical estrogens, essential fatty acids, and androgens define the sub-threshold range. These chemicals have measurable interaction with ERα, but with insufficient mechanistic potencies to be physiologically relevant as estrogens, and they do not produce clinically evident estrogenic responses by this MoA. The HRPT for the ERα agonist-mediated MoA would be between the supra-threshold and sub-threshold ranges of potency, within the range of substances that have intermediate mechanistic potency, e.g., between the low-potency and high-potency botanic estrogens. The HRPT could be approximated within this intermediate range if differences in mechanistic potency between the weaker and stronger botanical estrogens correlate with evidence for a lack of, versus the presence of clinically measurable ERα-mediated effects in humans.

### ERα agonist endpoints

For this prototype HRPT, the in vitro ERα transcription activation assay (ERαTA) and the in vivo uterotrophic assay were selected to provide data on mechanistic potency for ERα agonist-specific mechanisms. Because some chemicals act as pro-hormones, requiring metabolic conversion to an active metabolite of the parent molecule, it can be important to measure mechanistic estrogenic potency in the presence and absence of xenobiotic metabolizing systems. This can be accomplished either via in vitro pre-treatment with microsomal enzymes prior to a functional in vitro assay, such as the ERαTA, or by use of the in vivo uterotrophic assay in rodents. The rodent uterotrophic assay is optimized for sensitivity to estrogen e.g., by conducting the assay either in ovariectomized adults or in immature females during the narrow age window when the uterus is becoming sensitive to estrogens but levels of endogenous ERα agonists are still exceedingly low and progesterone levels elevated (Meijs-Roelofs et al. 1975). Exposure of the immature rodent uterus to exogenous ERα agonists prior to this window would induce the expression of progesterone receptors, resulting in down regulation of estrogenic responsiveness (Kraus and Katzenellenbogen 1993). To correlate estimates of mechanistic potency in these in vitro and in vivo systems with estrogen agonist effects of chemicals in humans, we used various clinical endpoints measured in human trials.

### In vitro ERα agonist mechanistic potencies

Table 1 shows relative mechanistic potency calculations for various substances based on equally effective concentrations (e.g., EC_50_ or EC_20_) relative to 17β-estradiol in widely used ERαTAs. The range of mechanistic potencies spans approximately six orders of magnitude, with 17α ethinyl estradiol equipotent to 17β-estradiol, diethylstilbestrol within a factor of 2, and the secondary endogenous estrogen estrone within two orders of magnitude less potent than 17β-estradiol or 17α ethinyl estradiol. The botanical estrogens range from two to six orders of magnitude less potent than 17β-estradiol, and androgens are approximately six orders of magnitude less potent. Zearalenone and 8-prenylnaringenin have in vitro mechanistic potencies similar to the secondary endogenous estrogens and several botanical estrogens, such as α-erythroidin, equol, coumestrol, genistein, zearalenol, and zearalenone have potencies just below that range. The cyclic siloxane octamethylcyclotetrasiloxane (D4) is no more potent than androgens. Progestins and progestogens are typically without detectable activity in ERαTA or rat uterotrophic assays (ICCVAM 2011). Some variability in potency estimates is expected and depends largely on methodology, especially for estimates based on affinity alone (e.g., Borgert et al. 2003).

**Table 1:** Relative potencies calculated^A^ from published human ERα transactivation results

Chemical	Mean relative potency	Median relative potency	Range	Data sources
Hormones				
17β-estradiol	1.0E+00			All sources
Estrone	3.5E−02	2.9E−02	1.0E−02 to 7.5E−02	B, K1, K2, K3, P, Y
Estriol	1.4E−01	8.3E−02	3.3E−02 to 4E−01	B, K1, K2, K3, P
Pharmaceuticals				
17α-estradiol	2.6E−02	2.6E−02	5.3E−03 to 4.7E−02	B, Y
17α-ethinyl estradiol	2.1E+00	1.3E+00	1.1E+00 to 5.7E+00	B, K1, K2, K3, P, Y
Diethylstilbestrol	2.0E+00	1.4E+00	2.5E−01 to 8.0E+00	B, C, G, K1, K2, K3, L, O
Tamoxifen	1.6E−05	8.3E−06	7.1E−07 to 4E−05	K1, K2, K3
Botanicals				
Equol	2.4E−03	3.6E−04	1.8E−04 to 1E−02	F, G, H, J, L, Q, S
Dehydroequol	NA	6.3E−05	NA	Q
Coumestrol	8.3E−03	1.0E−03	4.0E−05 to 8.0E−02	B, C, G, J, K1, K2, K3, L, P, S, U
Genistein	1.3E−03	4.5E−04	1.2E−05 to 1.0E−02	B, C, D, E, F, H, J, K1, K2, K3, L, N1, N2, O, P, Q, S, T, U, Y
Genistein glucuronide	NA	6.7E−07	NA	T
Daidzein	1.8E−04	4.7E−05	2.7E−06 to 9.7E−04	B, C, D, E, F, H, J, N1, N2, O, P, Q, S, T
Daidzein glucuronide	NA	6.7E−08	NA	T
*O*-DMA (daidzein metabolite)	NA	1.4E−04	NA	S
347-IF (daidzein metabolite)	6.1E-05	6.1E−05	2.8E−06 to 1.2E−04	L, S
674-IF (daidzein metabolite)	NA	6.0E−05	NA	L
Resveratrol	1.0E−04	1.0E−04	1.3E−07 to 2E−04	L, S
Resveratrol-3-*O*-SO_4_	NA	1.4E−05	NA	M
Biochanin A	2.2E−04	1.5E−04	2.5E−06 to 6.2E−04	C, D, E, F, H, J, P, S
Formononetin	2.4E−04	2.4E−04	9.6E−06 to 4.9E−04	D, E, F, J, S
Zearalenol	NA	7.0E−02	NA	C
Zearalenone	4.5E−01	3.4E−01	5.1E−03 to 1E+00	C, L, Y
Enterolactone	4.7E−05	2.1E−06	1.0E−07 to 1.4E−04	S, L, V
Enterodiol	NA	3.5E−07	NA	S
Sesamol	NA	2.0E−06	NA	V
Sesamin	3.3E−05	2.3E−05	2E−07 to 7.7E−05	H, J, V
Sesamolin	NA	6.7E−08	NA	V
Apigenin	9.7E−05	9.7E−05	3.4E−06 to 1.9E−04	J, S
Naringenin	6.1E−05	2.3E−05	1.9E−06 to 2E−04	C, H, J, S, W2
8-Prenylnaringenin (8-PN)	7.0E−02	8.6E−02	1E−02 to 1E−01	W1, W2, X1, X2
6-(l,l-dimethylallyl)naringenin	1.3E−02	1.7E−03	1E−03 to 1E−02	W1, W2, X2
8-PN-OH (metabolite of 8-PN)	2.1E−03	2.1E−03	1E−01 to 3.1E−03	X1, X2
8-PN-O (metabolite of 8-PN)	NA	1.4E−03	NA	X1
α-Erythroidin	1.1E−03	1.1E−03	1.0E−03 to 1.2E−03	R1, R2
β-Erythroidin	5.0E−04	5.0E−04	5E−04 to 5E−04	R1, R2
Androgens				
Testosterone	NA	7.1E−06	NA	B
Dihydrotestosterone	NA	9.6E−06	NA	Y
Methyltestosterone	NA	2.5E−06	NA	B
19-Nortestosterone	NA	4.7E−06	NA	B
Test case chemical				
D4	NA	5.0E-06	NA	I

Individual potency values for this table are found in electronic supplementary material

^A^Unless provided in the publication, Relative Potencies were derived by dividing an EC% value of the primary endogenous human estrogen, 17β-estradiol, by the equivalent EC% value of each chemical. EC% values were either extracted from tables or interpolated from figures. Where necessary, concentrations were adjusted to better approximate equivalent EC% values. *NA* not applicable

^B^Values derived from Table 4-1; ICCVAM 2011. [BG1Luc]

^C^Values derived from Table 3; Jefferson et al. 2002. [BG1Luc]

^D^Values derived from Table 2; Dornstauder et al. 2001. [YES]

^E^Values derived from Table 3; Beck et al. 2003. [YES]

^F^Values derived from Table 2; Pfitscher et al. 2008. [YES]

**Fig. 1:**
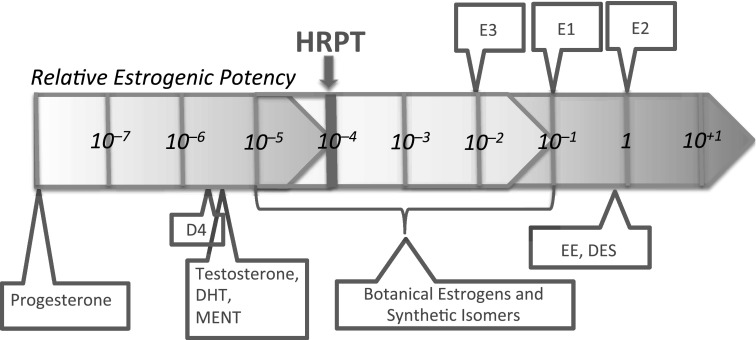
Human-relevant potency threshold for the ERα-agonist MoA

^G^Values derived from Fig. 1; Coldham et al. 1997. [YES]

^H^Values derived from Table 1; Breinholt and Larsen 1998. [YES]

^I^Values taken from Fig. 4; Quinn et al. 2007. [MCF-7]

^J^Values from Table 3; Procházková et al. 2017. [HeLa-9903]

^K^Values from Table 2; Gutendorf and Westendorf 2001

^L^Values from Fig. 3a RP ratios; Mueller et al. 2004. [Ishikawa EREα]

^M^Values from Table 1; Ruotolo et al. 2013. [YES]

^N^Values from Fig. 1; Chrzan and Bradford 2007. [N-1: MCF-7 / N-2: G-292]

^O^Values from Table 1; Chu et al. 2009. [YES-SRC-1]

^P^Values from Table 2; Escande et al. 2006. [HELN]

^Q^Values from Table 2; De Angelis et al. 2005. [HEC-1]

^R^Values from Figs. 2, 3; Djiogue et al. 2014. [R-1: MVLN / R-2: U2OS]

^S^Values from Table 1; Takeuchi et al. 2009. [CHO]

^T^Values from Table 1; Islam et al. 2015. [U2OS]

^U^Values from Table 2; Harris et al. 2005. [MCF-7]

^V^Values from Pianjing et al. 2011. [TD47-KBLuc]

^W^Values from Zierau et al. 2002. [W-1: YES / W-2: MVLN]

^X^Values from Zierau et al. 2004. [X-1: YES / X-2: MVLN]

^Y^Values from Table 1 (PC10, PC50), Yamasaki et al. 2002b. [HeLa229]

### In vivo ERα agonist mechanistic potencies

When measured in vivo with the uterotrophic assay, the same relative order of mechanistic potencies is generally observed among the groups of chemicals (Table 2). Endogenous estrogenic hormones and pharmaceutical estrogens are the most potent chemicals via ERα; 17β-estradiol, 17α-ethinylestradiol and diethylstilbestrol define the supra-threshold potency range and serve as positive controls and reference chemicals for this assay (Kanno et al. 2003). Although both aromatizable and non-aromatizable androgens increase uterine weight in rats, their uterotrophic action is antagonized by anti-androgens, with little inhibition by anti-estrogens except at very high doses (Beri et al. 1998; Armstrong et al. 1976; Schmidt et al. 1976; Schmidt and Katzenellenbogen 1979). Thus, androgens appear to exert a predominantly anabolic effect on the uterus rather than an estrogenic effect, which is consistent with our contention that their in vitro mechanistic potencies at ERα (approximately 5 × 10^−6^ relative to 17β-estradiol; see Table 1) are insufficient to be physiologically effective.

**Table 2:**
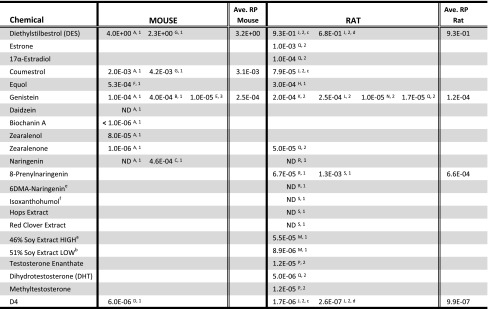
Uterotrophic relative potencies calculated* from values reported in the literature

*Unless provided in the publication, Relative Potencies (RP) were derived by dividing an EC% value (listed or estimated) of the reference chemical ^1,2,3^ by the equivalent EC% value (listed or estimated) of each test chemical. EC% values were either extracted from tables or interpolated from figures. Where necessary, concentrations were adjusted to better approximate equivalent EC% values. *ND* not different than control

^1^Estradiol-17β (E2) used as reference chemical for calculation of relative potencies

^2^Ethinyl estradiol-17α (EE) used as reference chemical for calculation of relative potencies

^3^Diethylstilbestrol (DES) used as reference chemical for calculation of relative potencies

^a^High content of genistin/genistein/glycitein

^b^Low content of genistin/genistein/glycitein

^A^Jefferson et al. (2002). CD-I immature mice; SQ injection; dose range 0.01–1000 000 ng/kg/day

^B^Lomnitski et al. (2003). Immature CD-I mice; SQ injection; 500 and 500,000 μg/kg/day

^C^Swankar et al. (2012). Ovariectomized Balb/cByJ mice; SQ injection; 5 mg/kg/day

^D^He et al. (2003). Ovariectomized B6C3F1 mice; oral dose; 1, 10, 50, 100, 250, 500, 1000 mg/kg

^E^Song et al. (1999). Immature B6D2F1 mice; oral gavage; 3 mg/day

^F^Selvarag et al. (2004). Ovariectomized age-matched C57BL/6 mice; SQ injection 0, 4, 8, 12, 20 mg/kg/day or in the diet 0, 500, 1000 ppm for 12 days

^G^Coldham et al. (1997). Prepubertal CFLP mice; SQ injection; DES doses of 0.5, 5, 50 ng; coumestrol doses of 1, 10, 100 μg

^H^Rachon et al. (2007). Ovariectomized SD rats; in diet; 400 mg/kg of chow

^J^McKim et al. (2001). Immature Sprague-Dawley^c^ and Fischer − 344^d^ rats; oral gavage; 10, 50, 100, 250, 500, 1000 mg/kg/day

^K^Yamasaki et al. (2002a). Immature Crj:CD(SD) rats; SQ injection; 0.6 ng/kg/day (EE) − 30 mg/kg/day (Genistein)

^L^Schmidt et al. (2006). Ovariectomized Wistar rats; oral dose by gastric tube; 100 mg/kg

^M^de Lima Toccafondo Vieira et al. (2008). Immature Wistar rats; oral gavage; doses 125, 300, 720, 1730, 4150 mg/kg/day

^N^Punt et al. (2013). Immature rats, SQ injection of 35 mg/kg/day

^O^Barlas et al. (2014). Immature Wistar rats; oral gavage; 1, 10, 100 mg/kg/day

^P^Yamasaki et al. 2003. Immature Crj:CD(SD) rats; SQ injection; 0.6 ng/kg/day–40 mg/kg/day

^Q^Yamasaki et al. (2002b). Immature Crj:CD(SD) rats; SQ injection; 0.2 ng/kg/day–200 mg/kg/day

^R^Keiler et al. (2015). Immature Lewis rats; SQ injection; 4 μg/kg/day–15 mg/kg/day, ^e^6-(l,l-dimethylallyl)naringenin

^S^Overk et al. (2008). Ovariectomized Sprague Dawley rats, 200 g body weight. 50 μg/kg/day E2; 4 mg/kg/day (8-PN); 4-400 mg/kg/day (extracts). Chemicals measured in plasma (< 0.5–3.7 ng/mL), liver (< 2–4.4 ng/g) and mammary gland (< 3–0.6 ng/g). ^f^Metabolite of 8-prenylnaringenin

Several botanical estrogens are more potent than androgens at ERα (Table 1), but unlike androgens, the uterotrophic effects of those tested are completely blocked by estrogen-receptor antagonists (Yamasaki et al. 2002b; Woods and Hughes 2003). Their uterotrophic potencies based on data from mouse and rat are generally consistent with or lower than their in vitro potencies, on the order of 10^−3^–10^−5^ relative to 17β-estradiol or 17α-ethinyl estradiol (Table 2; Woods and Hughes 2003). None of the botanical estrogens show greater in vivo mechanistic potency (Table 2) than in vitro (Table 1). 8-prenylnaringenin has the highest in vivo mechanistic potency (Table 2) of the botanical estrogens, but is more potent in vitro by an order of magnitude (Table 1). Although zearalenone appears to be the most potent botanical estrogen in vitro (Table 1), it is four orders of magnitude less potent in vivo (Table 2). Diethylstilbestrol has mechanistic potency 100–1000 times greater than those botanical estrogens in vivo. The uterotrophic potency of D4 is roughly 100 times lower than the least potent of the botanical estrogens, on the order of 10^−6^–10^−7^ relative to ethinyl estradiol in rats and 17β-estradiol in mice, but unlike the androgens, its uterotrophic activity is completely blocked by the pure estrogen antagonist ICI-182,780 and is absent in ERα-knockout mice (He et al. 2003).

## Comparison of mechanistic potencies to clinical endpoints

HRPTs can be estimated by comparing clinical data on the physiological effects in humans of chemicals that vary widely in their relative mechanistic potencies for ERα agonism. The estrogenic effects of 17β-estradiol, and 17α-ethinyl estradiol, are well known (Simpson and Santen 2015), producing consistent effects in humans, non-human primates, and in assays specific and sensitive for ERα agonism such as ERαTA and uterotrophic assays. The minor hormonal estrogens estrone and estriol have mechanistic potencies within two orders of magnitude of 17β-estradiol (Watson et al. 2008). Hence, chemicals that reproducibly exhibit in vitro and in vivo ERα agonist potencies within two orders of magnitude of 17β-estradiol would predictably produce estrogenic effects in human males and females. Of note, α-erythroidin, an alkaloid found in certain Chinese herbal medicines, appears to have a mechanistic potency within three orders of magnitude of 17β-estradiol (Table 1) and is reported to have abortifacient activity (Djioque et al. 2014).

Soy isoflavones, which are three to five orders of magnitude less potent than 17β-estradiol (Table 1), have been extensively evaluated and have shown no clear evidence of ERα-mediated estrogenic activity at high dietary levels of exposure or at higher doses when administered as post-menopausal hormone replacement in women (Cline et al. 2001; McCarty 2006; Woods and Hughes 2003; Bedell et al. 2014; EFSA 2015). EFSA (2015) concluded that the results of four epidemiological studies (2216 isoflavone users, total) and ten interventional controlled studies (816 participants, total) did not show an association between exposure to isoflavones-containing food supplements and adverse effects in breast, and that the majority of animal studies were negative. For uterus, EFSA (2015) found a general lack of statistically significant changes in endometrial thickness compared to control. A few histopathological effects and a few cases of endometrial hyperplasia were reported, but not cases of endometrial carcinoma. EFSA (2015) found that most of the animal studies that evaluated uterine effects were also negative.

Similarly, the results of a recent meta-analysis found that ingestion of soy protein or isoflavone, even at levels that greatly exceed the typical Japanese dietary intake, has no effect on reproductive hormone concentrations in men as measured by testosterone, sex-hormone binding globulin, free plasma testosterone and free androgen index (Hamilton-Reeves et al. 2010). Similarly, soy or soy isoflavones lack significant effects on circulating estrogen levels, sperm counts or sperm parameters, and there is no compelling evidence that soy intake produces erectile dysfunction or gynecomastia in men (Messina 2010). Vandenplas et al. (2014) found no strong evidence of adverse outcomes among published cross-sectional, case–control, cohort studies or clinical trials conducted in children fed soy-based infant formulas from 1909 to 2013, and which provided information on anthropometric growth, bone health (bone mineral content), immunity, cognition, and reproductive and endocrine functions. Infants fed soy-based infant formulas had similar levels of hemoglobin, serum protein, zinc and calcium concentrations and bone mineral content as children fed formulas based on cow’s milk or vegan human-milk substitutes (Vandenplas et al. 2014).

Some lignans and essential fatty acids have ERα agonist potencies similar to the lower-potency soy isoflavones based on binding affinities and cellular responses (Jin et al. 2013; Liu et al. 2004; Pianjing et al. 2011; Rose and Connolly 1989; Menendez et al. 2004). Although their agonistic interactions with ERα are detectable in the highly sensitive ERαTA (Table 1: enterolactone, enterodiol, sesamin, sesamol, sesamolin) and the in vivo uterotrophic assay (Penttinen-Damdimopoulou et al. 2009), their mechanistic potencies appear to be too low to produce overt physiological effects in either rodents or humans (Table 3). Beneficial effects attributed to dietary lignans and dietary supplements containing lignan extracts may be due to MoAs other than ERα agonism (Adolphe et al. 2010).

**Table 3:** Estrogenic efficacy of flaxseed/linoleic acid

Estrogenic endpoint	In vitro activity	In vivo effects	
		Rodents	Women
Cell proliferation (MCF-7/MDA-MB-231)	✓ [1]		
Cell proliferation (non-breast cell lines)	− [1]		
Alkaline phosphatase activity	− [2]		
Prog-RmRNA induction	✓ [2]		
Era binding	✓ [2]		
Erp binding	✓ [2]		
Uterine columnar luminal epithelial		✓ [5]	
Enhanced responses to low-dose E2		− [5]	
Uterine cell proliferation		− [5]	
Enhanced responses to low-dose E2		− [5]	
Serum El, El-sulfate, E2			− [3]
Serum deoxypyridinoline (free-DPD)			− [3]
Bone-specific alkaline phosphatase			− [3]
Urinary estrogen metabolites altered			✓ [3]
Blood E2			− [4]
Blood FSH			− [4]
Vaginal epithelial thickness			− [4]
Endometrial thickness			− [4]
Kupperman Index (paired *t* test)			✓ [4]
Kupperman Index (Anova)			− [4]

*✓* Activity; *–* No Activity; [1] Rose and Connolly (1989); [2] Liu et al. (2004); [3] Brooks et al. (2004); [4] Colli et al. (2012); [5] Sacco et al. (2012)

Studies in non-human primates are generally consistent with the human data. Although changes in maternal and fetal 17β-estradiol and testosterone levels and proliferation of Leydig cells have been reported in some studies with non-human primates administered high doses of soy isoflavones, clear estrogenic effects have generally not been observed (Cline et al. 1996; Woods and Hughes 2003; Wood et al. 2006; EFSA 2015). Differences in dose or bioavailability seem inadequate to fully explain the contrasting estrogenic effects of soy isoflavones in rodents versus non-human primates, as a recent pharmacokinetic investigation indicates that genistein administration produces circulating blood levels in rhesus monkeys comparable to or higher than those that produce adverse reproductive effects in rodents (Doerge et al. 2016). Instead, the difference is likely due to a selectivity of soy isoflavones for the ERβ subtype in humans and non-humans primates (Jiang et al. 2013).

Important differences in pharmacokinetic parameters (Soukup et al. 2016) suggest that humans would be slightly less sensitive than rats and substantially less sensitive than mice to the effects of an equivalent internal dose of soy isoflavones. In humans, the more highly estrogenic aglycone derivatives represented a very small proportion of the total plasma isoflavones, which was a slightly lower proportion than seen in rats overall and substantially lower than in mice. The proportion of conjugated isoflavones was very high in humans, similar to rats, and greater than in mice. All male and female rats and mice converted daidzein to the more estrogenic *S*-equol, whereas only 36% of women and 20% of men showed this capacity (Soukup et al. 2016). These factors suggest that mechanistic potencies for the various isoflavones will not be underestimated from results of transactivation assays and rodent uterotrophic assays.

Despite the apparent lack of ERα agonist-mediated estrogenic activity in humans, botanical estrogens and isoflavones are now used as hormonal replacement therapy by millions of post-menopausal women. Initially, therapeutic benefits were questionable. Sirtori et al. (2005) concluded that the clinical epidemiological data did not support claims of therapeutic effects by soy isoflavones, even at high levels of intake, and that the physiological effects of phytoestrogens appeared to be insignificant in humans. Although intra-individual and interspecies differences in intestinal conversion and bioavailability of isoflavones and their derivatives (Woods and Hughes 2003) account for some of this variability, mechanistic potency also plays an important role. With recognition of the importance of standardizing botanical preparations to the most potent constituents (de Lima Toccafondo Vieira et al. 2008; Messina 2014) have come an increasing proportion of studies that do show efficacy. Table 4 summarizes extensive reviews published since Sirtori’s (2005) analysis that report some efficacy of soy isoflavones among 60 clinical trials and more than 25,000 subjects, but no attributable adverse effects (Messina 2014; EFSA 2015).

**Table 4:** Human effects of soy foods and soy isoflavones

Estrogen-related endpoint	Dose/subjects	Result	Receptor involvement
Messina (2014)			
Bone mineral density	24,403 PM Asian women/>10 g/day soy protein	No effect	ERα, ERB
Hip fracture risk reduction		28–37%	ERα, ERβ; Calcium
Hip fracture risk reduction	35,241 PM Asian women/>10 g/day soy protein	21–36%	ERα, ERβ; calcium
Hip fracture risk reduction	307 PM women	56%	ERα, ERβ; calcium
Reduced hot flashes	60 clinical trials/various products	Mixed	ERα, ERβ
Reduced hot flashes	> 50 mg/day total isoflavones for > 12 weeks	~ 50%	ERα, ERβ
Reduced hot flashes	> 19 mg/day genistein dose threshold	29%	ERα, ERβ
Reduced hot flashes	< 19 mg/day genistein dose threshold	12%	ERα, ERβ
EFSA (2015)			
Breast tissue morphology		No adverse effects	ERα, ERβ
Breast tissue; disease progression	2216 women: 4 epidemiology studies of high isoflavone intake 816 women: intervention trials of soy food supplements	No adverse effects	ERα, ERβ
Uterine endometrial thickness		No change	ERα, ERβ

Standardizing preparations to the most potent estrogenic constituents has not increased the incidence of adverse side effects typically associated with pharmaceutical estrogens. This improved efficacy without adverse ERα-mediated proliferative side effects may be explained by the selectivity of isoflavones for ERβ relative to ERα (McCarty 2006). For several major isoflavones, the affinities for ERα and ERβ are similar, but the relative effects on transactivation are much different (Pfischer et al. 2008; Dornstauder et al. 2001). Thus, the potency of bioavailable isoflavones via ERβ approaches that of pharmaceutical estrogens and produces physiologically detectable effects, while their potency via ERα is lower and physiologically less significant in humans.

## Proposed HRPT for the ERα agonist MoA

Relative mechanistic potencies of botanical isoflavones, as reflected in the ERα-transactivation and uterotrophic potencies of isoflavones used to standardize botanical estrogenic supplements, would thus appear to define a potency threshold below which adverse effects in humans are unlikely. Based on in vitro (Table 1) and in vivo (Table 2) relative potency values for coumestrol, equol, and genistein, and comprehensive reviews by the UK Committee on Toxicity of Chemicals in Food, Consumer Products and the Environment (Woods and Hughes 2003) and by the European Food Safety Authority, EFSA (EFSA 2015), a conservative estimate of this potency threshold would be in the range of 1.0E−04 relative to the potency of 17β-estradiol (Tables 1, 2). It would seem improbable for chemicals with relative potencies below this level to exhibit physiological effects via ERα in humans. Indeed, we have found no literature documenting bona fide ERα agonist effects in humans produced by substances with ERα agonist potencies below this level. Figure 1 shows these ranges of potency graphically.

It is important to recognize that our proposed HRPT for the ERα agonist MoA does not depend on an assumption that isoflavones pose no health risks. Our proposed HRPT for this MoA is below the potency of the more potent botanical estrogens, including genistein, equol, and coumestrol (Table 1), which are thought to be responsible for any potential estrogenic health effects, to the extent that they exist. The fact that high isoflavone intakes have not been shown to produce clinically measurable adverse effects in adults or infants, as discussed above, argues for the conservativeness of our proposed HRPT.

### Sub-threshold substances: case study of D4 (octamethyltetrasiloxane)

A careful examination of existing data indicates that ERα agonists with sub-HRPT potencies are weak partial agonists. Partial agonists bind in the agonist binding site of the receptor but are less effective at stabilizing the stimulated (active) conformation of the receptor, and therefore are incapable of producing the same maximal response as a full agonist. A partial agonist in isolation will look like an agonist. A partial agonist in combination with a concentration of a full agonist that produces a lower-than-maximal effect of the partial agonist in isolation will act additively with the full agonist. When the full agonist is present at concentrations that produce effects greater than the maximal effect of the partial agonist in isolation, the partial agonist will act as a competitive antagonist of the full agonist (Matthews 1993), as occurs with genistein in mice (Erlandsson et al. 2005). Mechanistic potency dictates these relationships, as illustrated by the observation that transactivation in the presence of estradiol was only evident for the more highly potent botanical estrogens evaluated (Harris et al. 2005).

In the intact organism, endocrine systems are highly regulated; introducing a partial agonist with sub-threshold mechanistic potency into the system may result in adjustments by the system such that there will be a little or no measurable change. Thus, logical extension of classical pharmacological theory leads to the prediction that when the endogenous estrogen is present at low concentrations, a weak partial agonist may transiently increase the total estrogenic activity, but negative feedback will react to decrease endogenous estrogenic activity sufficient to maintain a consistent estrogenic tone. However, empirical demonstration of this phenomenon would be difficult for weak partial agonists because the expected feedback response in circulating endogenous estrogen would likely remain within normal ranges. Consistent with this explanation, as mentioned previously herein, soy and soy isoflavones lack significant effects on circulating estrogen levels in men, and even very high doses do not consistently produce measurable changes in non-human primates.

When the endogenous estrogen is present at concentrations that produce a substantial or maximal response, the partial agonist may competitively inhibit the actions of the endogenous (full) agonist, resulting in an increased production of endogenous agonist. There may also be adjustments in the levels of available receptors as the result of these internal regulatory systems. It is, therefore, implausible that partial agonists with sub-threshold mechanistic potencies could produce measurable effects on the intact system. Only those agonists with sufficiently high mechanistic potencies can achieve concentrations sufficient to overcome regulatory feedback or other systems necessary to produce more than transiently measurable effects.

Furthermore, with or without contributions from other components of the system, e.g., co-activators, full agonists are often capable of producing more than one stimulated conformation of their receptor. Different stimulated conformations may be responsible for producing different physiological effects. Weak partial agonists may not be capable of producing the full spectrum of stimulated conformations, and therefore, may not be capable of producing a full range of physiological effects.

D4 (octamethylcyclotetrasiloxane), a cyclic siloxane used in the production of silicone polymers, is a clear example corroborating that partial ERα agonism below the threshold mechanistic potency proposed here (1E-04 relative to 17β-estradiol or pharmaceutical estrogens) renders a substance incapable of producing measurable effects on the intact estrogen system. Consistent with this conclusion, D4 was given a bioactivity score of zero in US EPA’s estrogen expert system (EDSP21 Dashboard 2017).^2^ The mechanistic potency of D4 via ERα is in the range of 5E-06–2E-07 relative to 17β-estradiol or pharmaceutical estrogens in vitro (Table 1) and in vivo (Table 2). At its maximum achievable vapor concentration (700 ppm; Quinn et al. 2007) and oral doses (1000 mg/kg/day; McKim et al. 2001; He et al. 2003), D4 only partially displaces 17β-estradiol binding from ERα (He et al. 2003) but not from ERβ (Quinn et al. 2007). Its weak uterotrophic activity and weak partial antagonism of full uterotrophism by 17β-estradiol or 17α-ethinyl estradiol occurs at high air concentrations (700 ppm; Quinn et al. 2007) and oral doses (1000 mg/kg/day; McKim et al. 2001; He et al. 2003), but is absent by the oral route of exposure in ERα-knockout mice (He et al. 2003) and in some rat studies (Lee et al. 2015).

If the level of mechanistic potency exhibited by D4 were sufficient to produce physiologically relevant ERα-mediated effects in intact animals, alterations in a variety of estrogen-sensitive endpoints could be observed (Neal et al. 2017). As predicted by its sub-threshold mechanistic potency, however, D4 fails to produce an estrogenic pattern of effects in either chronic repeated-dose (Dekant et al. 2017) or multigenerational reproduction (Siddiqui et al. 2007) toxicity tests in rats. Effects in male offspring are conspicuously absent from D4’s effect profile, including alterations of secondary sex organ weights and development (e.g., delayed preputial separation; reduced testicular weight; reduced fertility). The effects of D4 on reproductive tissues and function that have been observed occur only at high doses and appear to be produced by a rodent-specific MoA involving interruption of the pre-ovulatory LH surge (Dekant et al. 2017), not directly by an ERα agonist-mediated MoA.

## Discussion

The HRPT derived here for the ERα agonist MoA can be used to evaluate chemicals according to the criteria for identification of EDCs proposed by the European Commission, which requires establishing the biological plausibility of a causal link between the adverse effects of a chemical and an endocrine MoA. For the ERα agonist MoA, plausibility would require that the adverse effects of the chemical are among those known to be produced by ERα agonists in chronic studies using animals with intact endocrine systems (Neal et al. 2017) or in humans, and that their mechanistic potency via the ERα agonist MoA exceeds the HRPT of 1E-04 relative to the supra-threshold reference ERα agonists, e.g., 17β-estradiol or 17α-ethinyl estradiol.

The adverse effects of chemicals with supra-threshold ERα agonist mechanistic potencies (i.e., in the range of the endogenous hormones and pharmaceutical estrogens) can be considered to have a high likelihood of being caused by an ERα MoA. The biological plausibility of a causal link between adverse effects consistent with an ERα agonist MoA and interactions with the ERα system can be considered possible, on a precautionary basis, for chemicals with mechanistic potencies just above the HRPT, i.e., in the range of 1E−02– 1E−04. For those chemicals, further experimental investigation could be used to resolve whether the MoA for adverse effects is mediated via an ERα agonist MoA. Experiments that employ counterfactual study designs and methodologies are most useful for resolving such uncertainties (reviewed in Borgert et al. 2011, supplemental materials). Chemicals with mechanistic potencies via the ERα agonist MoA below the HRPT should be eliminated from consideration as EDCs via the ERα agonist MoA. For such chemicals, the likelihood of a biologically plausible causal link between adverse effects and the ERα agonist MoA is so remote, and the ability to provide scientifically valid evidence for such a link is so limited, that use of this threshold can be considered a precautionary basis for regulatory decision-making.

The methodology used here to derive the HRPT for the ERα agonist MoA serves as a model by which other potency thresholds can be developed and provides a template for developing HRPTs useful in scientific and regulatory decision-making for any particular MoA for which reliable assays exist that measure mechanistic potency, and for which physiologically relevant correlates can be made by empirical comparisons. HRPTs developed according to this methodology would be applicable not only for identification of EDCs by the EC Criteria, but would be generally useful for hazard identification by any endocrine or non-endocrine MoA that relies on specific molecular events that are subject to the fundamentals of receptor, enzyme, and transport kinetics employed here. The HRPT concept is applicable to any assessment that relies on MoA, such as defining common assessment groups for component-based mixtures risk assessments and for delineating the domain of applicability for Adverse Outcome Pathways (Ankley et al. 2009; AOP Wiki 2017), which are defined according to specific MoAs.

Depending upon the availability of reliable assays for measuring the mechanistic potencies by which chemicals may interact with key molecular components comprising a specific MoA, HRPTs could be developed for MoAs encompassing any type of activity with a wide variety of receptors, enzymes, chaperones, transporters, etc. Within our group, the methodology is currently being used to develop an HRPT for the ERβ agonist MoA. Decision-making for EDCs, however, could proceed for individual endocrine MoAs based on the existing information and HRPTs developed as described herein. There is no scientific reason to delay such decision-making until potency data are available for all molecular components of endocrine-signaling systems.

Consistent with the inception of the HRPT concept (Borgert et al. 2012), the HRPT developed here for the ERα agonist MoA is applicable beyond identification of EDCs by the EC Criteria. Specifically, chemicals with sub-threshold ERα agonist potencies should be considered ineligible for inclusion in common assessment groups for chemical mixtures. As explained herein, chemicals with such weak mechanistic potency are unable to influence endogenous estrogenic signaling due to their inability to overcome feedback mechanisms controlling the functional state of the estrogenic system. Chemicals with sub-threshold mechanistic potencies are mechanistically and physiologically indistinguishable via the ERα-signaling pathway from the 10^5^- to 10^9^-fold molar excess of endogenous small molecules present naturally in the bloodstream, many of which have similarly weak interactions with components of the endocrine system (reviewed in Borgert et al. 2013, and citations therein).

## Electronic supplementary material

Below is the link to the electronic supplementary material.

Supplementary material 1 (PDF 103 KB)

## Footnotes

1 It can be argued that indirect mechanisms of interaction with the endocrine system can also alter endocrine function. This is not in dispute, but falls outside the EDC definition. For example, dibromochloropropane has overtly anti-androgenic effects as a consequence of its lethality to testicular cells. This MoA would not fit the EDC definition because the destruction of testicular cells, and the antecedent effects, including effects on androgen synthesis, are causally dependent on cellular toxicity rather than on an interaction with the androgen system. Similar logic prevents us from affixing the EDC label to hepatotoxicants, such as ethanol, which at high levels can alter the processing of many important biological molecules, including steroid hormones.

2 The EDSP-21 estrogen expert system is a high-throughput suite of 18 assays that has been validated against in vivo estrogenic effects and is designed to prioritize chemicals for more comprehensive in vivo endocrine screening using information on mechanistic potency and cytotoxicity.
