# Supplementary material for: Human-relevant potency threshold (HRPT) for ERα agonism
Source: Arch Toxicol. 2018 Apr 9;92(5):1685–702. doi: 10.1007/s00204-018-2186-z (PMC5962616; doi:10.1007/s00204-018-2186-z)
Supplement: Supplementary file 1 — Supplementary material 1 (PDF 103 KB) [file 204_2018_2186_MOESM1_ESM.pdf]

SUPPLEMENT to Table 1: Relative Potencies Calculated[A] from Published Human ERα Transactivation Results

Chemical	EC_{50} (M) BG1Luc [B]	*Relative Potency BG1Luc [B]*	EC_{50} (M) BG1Luc [C]	*Relative Potency BG1Luc [C]*	EC_{50} (M) YES [D]	*Relative Potency YES [D]*	EC_{50} (M) YES [E]	*Relative Potency YES [E]*	EC_{50} (M) YES [F]	*Relative Potency YES [F]*	EC_{50} (M) YES [G]	*Relative Potency YES [G]*	EC_{50} (M) YES [H]	*Relative Potency YES [H]*
Hormones														
17β-Estradiol	8.4E-12	*1.0E+00*	8.4E-12	*1.0E+00*	4.1E-10	*1.0E+00*	6.8E-10	*1.0E+00*	2.6E-10	*1.0E+00*	9.0E-11	*1.0E+00*	2.3E-11	*1.0E+00*
Estrone	2.6E-10	*3.3E-02*												
Estriol	2.6E-10	*3.3E-02*												
Pharmaceuticals														
17α-Estradiol	1.5E-09	*5.4E-03*												
17α-Ethinyl Estradiol	7.3E-12	*1.1E+00*												
Diethylstilbestrol	3.3E-11	*2.5E-01*	1.8E-11	*4.6E-01*							2.8E-10	*3.2E-01*		
Tamoxifen														
Botanicals														
Equol									2.2E-07	*1.2E-03*	5.0E-07	*1.8E-04*	1.0E-07	*2.3E-04*
Dehydroequol														
Coumestrol	8.8E-08	*9.5E-05*	1.9E-08	*4.4E-04*							7.6E-08	*1.2E-03*		
Genistein	2.7E-07	*3.1E-05*	7.0E-07	*1.2E-05*	9.0E-07	*4.6E-04*	8.8E-07	*7.7E-04*	3.9E-07	*6.7E-04*			5.0E-07	*4.6E-05*
Genistein Glucuronide														
Daidzein	8.7E-07	*9.6E-06*	2.1E-06	*4.1E-06*	1.0E-05	*4.1E-05*	1.3E-05	*5.2E-05*	7.4E-06	*3.5E-05*			8.5E-06	*2.7E-06*
Daidzein Glucuronide														
O-DMA (Daidzein metabolite)														
347-IF (Daidzein metabolite)														
674-IF (Daidzein metabolite)														
Resveretrol														
Resveretrol-3-O-SO_4														
Biochanin A			7.4E-07	*1.1E-05*	9.5E-07	*4.3E-04*	1.1E-06	*6.2E-04*	6.7E-07	*3.9E-04*			9.2E-06	*2.5E-06*
Formononetin					2.0E-06	*2.1E-04*	1.4E-06	*4.9E-04*	1.1E-06	*2.4E-04*				
Zearalenol			1.2E-10	*7.0E-02*										
Zearalenone			1.7E-09	*5.1E-03*										
Enterolactone														
Enterodiol														
Sesamol														
Sesamin													3.0E-07	*7.7E-05*
Sesamolin														
Apigenin														
Naringenin			4.5E-06	*1.9E-06*									3.0E-07	*7.7E-05*
8-Prenylnaringenin (8-PN)														
6-(1,1-dimethylallyl)naringenin														
8-PN-OH (metabolite of 8-PN)														
8-PN-O (metabolite of 8-PN)														
α-Erythroidin														
β-Erythroidin														
Androgens														
Testosterone	1.2E-06	*7.11E-06*												
Dihydrotestosterone														
Methyltestosterone	3.3E-06	*2.5E-06*												
19-Nortestosterone	1.8E-06	*4.7E-06*												
Test Case Chemical														
D4														

Chemical	EC_{20} (M) MCF-7 [I]	*Relative Potency MCF-7 [I]*	*Relative Potency HeLa-9903 [J]*	*Relative Potency MVLN [K-1]*	*Relative Potency HGELN [K-2]*	*Relative Potency MCF-7 [K-3]*	EC_{50} (EREα) Ishikawa [L]	*Relative Potency Ishikawa [L]*	*Relative Potency YES [M]*	EC_{50} (M) MCF-7 [N-1]	*Relative Potency MCF-7 [N-1]*	EC_{50} G-292 [N-2]	*Relative Potency G-292 [N-2]*	EC_{50} (M) YES-SRC-1 [O]	*Relative Potency YES-SRC-1 [O]*
Hormones															
17β-Estradiol	5.0E-11	*1.0E+00*	*1.0E+00*	*1.0E+00*	*1.0E+00*	*1.0E+00*	1.0E+00	*1.0E+00*	*1.0E+00*	1.1E-10	*1.0E+00*	5.0E-10	*1.0E+00*	5.31E-10	*1.0E+00*
Estrone				*1.0E-02*	*5.6E-02*	*1.0E-02*									
Estriol				*8.3E-02*	*4.0E-01*	*7.1E-02*									
Pharmaceuticals															
17α-Estradiol															
17α-Ethinyl Estradiol				*1.3E+00*	*5.7E+00*	*1.3E+00*									
Diethylstilbestrol				*1.3E+00*	*8.0E+00*	*2.5E+00*	2.0E+00	*2.0E+00*						3.6E-10	*1.5E+00*
Tamoxifen				*8.3E-06*	*7.1E-07*	*4.0E-05*									
Botanicals															
Equol			*3.6E-04*				1.0E-02	*1.0E-02*							
Dehydroequol															
Coumestrol			*1.3E-03*	*1.3E-04*	*1.0E-03*	*1.1E-04*	8.0E-02	*8.0E-02*							
Genistein			*4.5E-04*	*1.3E-04*	*8.0E-04*	*1.3E-05*	8.0E-03	*8.0E-03*		3.3E-07	*3.3E-04*	6.6E-07	*7.6E-04*	3.6E-07	*1.5E-03*
Genistein Glucuronide															
Daidzein			*1.5E-04*							6.3E-07	*1.7E-04*	1.6E-05	*3.2E-05*	5.5E-07	*9.7E-04*
Daidzein Glucuronide															
O-DMA (Daidzein metabolite)															
347-IF (Daidzein metabolite)							1.2E-04	*1.2E-04*							
674-IF (Daidzein metabolite)							6.0E-05	*6.0E-05*							
Resveretrol							2.0E-04	*2.0E-04*							
Resveretrol-3-O-SO_4									*1.4E-05*						
Biochanin A			*8.9E-05*												
Formononetin			*2.4E-04*												
Zearalenol															
Zearalenone							3.4E-01	*3.4E-01*							
Enterolactone							1.4E-04	*1.4E-04*							
Enterodiol															
Sesamol															
Sesamin			*2.3E-05*												
Sesamolin															
Apigenin			*1.9E-04*												
Naringenin			*2.3E-05*												
8-Prenylnaringenin (8-PN)															
6-(1,1-dimethylallyl)naringenin															
8-PN-OH (metabolite of 8-PN)															
8-PN-O (metabolite of 8-PN)															
α-Erythroidin															
β-Erythroidin															
Androgens															
Testosterone															
Dihydrotestosterone															
Methyltestosterone															
19-Nortestosterone															
Test Case Chemical															
D4	1.0E-05	*5.0E-06*													

Chemical	EC_{50} (M) HELN-ERα [P]	*Relative Potency HELN-ERα [P]*	EC_{50} (M) HEC-1 [Q]	*Relative Potency HEC-1 [Q]*	*Relative Potency MVLN [R-1]*	*Relative Potency U2OS [R-2]*	EC_{50} (M) CHO [S]	*Relative Potency CHO [S]*	*Relative Potency U2OS [T]*	EC_{50} (M) MCF-7 [U]	*Relative Potency MCF-7 [U]*	EC_{50} (M) T47D-KBLuc [V]	*Relative Potency T47D-KBLuc [V]*
Hormones													
17β-Estradiol	1.70E-11	*1.0E+00*	2.0E-10	*1.0E+00*	*1.0E+00*	*1.0E+00*	2.5E-12	*1.0E+00*	*1.0E+00*	6.4E-11	*1.0E+00*	2.0E-12	*1.0E+00*
Estrone	6.6E-10	*2.6E-02*											
Estriol	1.6E-10	*1.1E-01*											
Pharmaceuticals													
17α-Estradiol													
17α-Ethinyl Estradiol	8.0E-12	*2.1E+00*											
Diethylstilbestrol													
Tamoxifen													
Botanicals													
Equol			2.0E-07	*1.0E-03*			4.6E-09	*5.4E-04*					
Dehydroequol			3.2E-06	*6.3E-05*									
Coumestrol	1.6E-08	*1.1E-03*					4.5E-10	*5.6E-03*		1.6E-06	*4.0E-05*		
Genistein	3.8E-08	*4.5E-04*	2.0E-08	*1.0E-02*			2.3E-09	*1.1E-03*	*1.6E-04*	4.6E-07	*1.4E-04*		
Genistein Glucuronide									*6.7E-07*				
Daidzein	1.5E-07	*1.1E-04*	2.5E-07	*8.0E-04*			2.4E-08	*1.0E-04*	*3.4E-05*				
Daidzein Glucuronide									*6.7E-08*				
O-DMA (Daidzein metabolite)							2.4E-08	*1.0E-04*					
347-IF (Daidzein metabolite)							8.8E-07	*2.8E-06*					
674-IF (Daidzein metabolite)													
Resveretrol							2.0E-05	*1.3E-07*					
Resveretrol-3-O-SO_4													
Biochanin A	8.2E-08	*2.1E-04*					2.1E-07	*1.2E-05*					
Formononetin							2.6E-07	*9.6E-06*					
Zearalenol													
Zearalenone													
Enterolactone							1.2E-06	*2.1E-06*				2.0E-05	*1.0E-07*
Enterodiol							7.2E-06	*3.5E-07*					
Sesamol												1.0E-06	*2.0E-06*
Sesamin												1.0E-05	*2.0E-07*
Sesamolin												3.0E-05	*6.7E-08*
Apigenin							7.4E-07	*3.4E-06*					
Naringenin							9.9E-07	*2.5E-06*					
8-Prenylnaringenin (8-PN)													
6-(1,1-dimethylallyl)naringenin													
8-PN-OH (metabolite of 8-PN)													
8-PN-O (metabolite of 8-PN)													
α-Erythroidin					*1.0E-03*	*1.2E-03*							
β-Erythroidin					*5.0E-04*	*5.0E-04*							
Androgens													
Testosterone													
Dihydrotestosterone													
Methyltestosterone													
19-Nortestosterone													
Test Case Chemical													
D4													

Chemical	EC_{50} (M) YES [W-1]	*Relative Potency YES [W-1]*	EC_{50} (M) MVLN [W-2]	*Relative Potency MVLN [W-2]*	EC_{50} (M) YES [X-1]	*Relative Potency YES [X-1]*	EC_{50} (M) MVLN [X-1]	*Relative Potency MVLN [X-2]*	PC_{10} (M) HeLa229 [Y]	*Relative Potency HeLa229 [Y]*	*Mean Relative Potency*	*Median Relative Potency*	*Range*	Data Sources	MIN	MAX	Molar Conversion Calculations[C] MW	Values	Molar Conversion
Hormones																			
17β-Estradiol	2.5E-09	*1.0E+00*	1.0E-09	*1.0E+00*	2.5E-09	*1.0E+00*	1.0E-09	*1.0E+00*	1.0E-11	*1.0E+00*	*1.0E+00 (Reference Chemical)*			All Sources			272.382	2.30E-06	8.44E-12
Estrone									1.3E-10	*7.5E-02*	*3.5E-02*	*2.9E-02*	1.0E-02 – 7.5E-02	B, K1, K2, K3, P, Y	1.0E-02	7.5E-02			
Estriol											*1.4E-01*	*8.3E-02*	3.3E-02 – 4E-01	B, K1, K2, K3, P	3.3E-02	4.0E-01			
Pharmaceuticals																			
17α-Estradiol									2.1E-10	*4.7E-02*	*2.6E-02*	*2.6E-02*	5.3E-03 – 4.7E-02	B, Y	5.4E-03	4.7E-02			
17α-Ethinyl Estradiol									9.0E-12	*1.1E+00*	*2.1E+00*	*1.3E+00*	1.1E+00 – 5.7E+00	B, K1, K2, K3, P, Y	4.7E-02	5.7E+00			
Diethylstilbestrol											*2.0E+00*	*1.4E+00*	2.5E-01 – 8.0E+00	B, C, G, K1, K2, K3, L, O	2.5E-01	8.0E+00	268.35	4.90E-06	1.83E-11
Tamoxifen											*1.6E-05*	*8.3E-06*	7.1E-07 – 4E-05	K1, K2, K3	7.1E-07	4.0E-05			
Botanicals																			
Equol											*2.4E-03*	*3.6E-04*	1.8E-04 – 1E-02	F, G, H, J, L, Q, S	1.8E-04	2.0E-02			
Dehydroequol											*NA*	*6.3E-05*	NA	Q					
Coumestrol											*8.3E-03*	*1.0E-03*	4.0E-05 – 8.0E-02	B, C, G, J, K1, K2, K3, L, P, S, U	4.0E-05	8.0E-02	268.224	5.20E-03	1.94E-08
Genistein									3.3E-08	*3.0E-04*	*1.3E-03*	*4.5E-04*	1.2E-05 – 1.0E-02	B, C, D, E, F, H, J, K1, K2, K3, L, N1, N2, O, P, Q, S, T, U, Y	1.2E-05	1.0E-02	270.241	1.90E-01	7.03E-07
Genistein Glucuronide											*NA*	*6.7E-07*	NA	T					
Daidzein											*1.8E-04*	*4.7E-05*	2.7E-06 – 9.7E-04	B, C, D, E, F, H, J, N1, N2, O, P, Q, S, T	2.7E-06	9.7E-04	254.23	5.20E-01	2.05E-06
Daidzein Glucuronide											*NA*	*6.7E-08*	NA	T					
O-DMA (Daidzein metabolite)											*NA*	*1.4E-04*	NA	S					
347-IF (Daidzein metabolite)											*6.1E-05*	*6.1E-05*	2.8E-06 – 1.2E-04	L, S	2.8E-06	1.2E-04			
674-IF (Daidzein metabolite)											*NA*	*6.0E-05*	NA	L					
Resveretrol											*1.0E-04*	*1.0E-04*	1.3E-07 – 2E-04	L, S	1.3E-07	2.0E-04			
Resveretrol-3-O-SO_4											*NA*	*1.4E-05*	NA	M					
Biochanin A											*2.2E-04*	*1.5E-04*	2.5E-06 – 6.2E-04	C, D, E, F, H, J, P, S	2.5E-06	6.2E-04	284.26	2.10E-01	7.39E-07
Formononetin											*2.4E-04*	*2.4E-04*	9.6E-06 – 4.9E-04	D, E, F, J, S	9.6E-06	4.9E-04			
Zearalenol											*NA*	*7.0E-02*	NA	C			322.401	3.90E-05	1.21E-10
Zearalenone									1.0E-11	*1.0E+00*	*4.5E-01*	*3.4E-01*	5.1E-03 – 1E+00	C, L, Y	5.1E-03	1.0E+00	318.364	5.30E-04	1.66E-09
Enterolactone											*4.7E-05*	*2.1E-06*	1.0E-07 – 1.4E-04	S, L, V	1.0E-07	1.4E-04			
Enterodiol											*NA*	*3.5E-07*	NA	S					
Sesamol											*NA*	*2.0E-06*	NA	V					
Sesamin											*3.3E-05*	*2.3E-05*	2E-07 – 7.7E-05	H, J, V	2.0E-07	7.7E-05	272.257	1.22E+00	4.48E-06
Sesamolin											*NA*	*6.7E-08*	NA	V					
Apigenin											*9.7E-05*	*9.7E-05*	3.4E-06 – 1.9E-04	J, S	3.4E-06	1.9E-04			
Naringenin	NR	*NR*	5.0E-06	*2.0E-04*							*6.1E-05*	*2.3E-05*	1.9E-06 – 2E-04	C, H, J, S, W2	1.9E-06	2.0E-04			
8-Prenylnaringenin (8-PN)	3.5E-08	*7.1E-02*	1.0E-07	*1.0E-02*	2.5E-08	*1.0E-01*	1.0E-08	*1.0E-01*			*7.0E-02*	*8.6E-02*	1E-02 – 1E-01	W1, W2, X1, X2	1.0E-02	1.0E-01			
6-(1,1-dimethylallyl)naringenin	1.5E-06	*1.7E-03*	1.0E-07	*1.0E-02*			1.0E-06	*1.0E-03*			*1.3E-02*	*1.7E-03*	1E-03 – 1E-02	W1, W2, X2	1.0E-03	1.0E-02			
8-PN-OH (metabolite of 8-PN)					8.0E-07	*3.1E-03*	1.0E-06	*1.0E-03*			*2.1E-03*	*2.1E-03*	1E-01 – 3.1E-03	X1, X2	1.0E-03	3.1E-03			
8-PN-O (metabolite of 8-PN)					1.8E-06	*1.4E-03*					*NA*	*1.4E-03*	NA	X1					
α-Erythroidin											*1.1E-03*	*1.1E-03*	1.0E-03 – 1.2E-03	R1, R2	1.0E-03	1.2E-03			
β-Erythroidin											*5.0E-04*	*5.0E-04*	5E-04 – 5E-04	R1, R2	5.0E-04	5.0E-04			
Androgens																			
Testosterone											*NA*	*7.1E-06*	NA	B					
Dihydrotestosterone									1.0E-06	*9.6E-06*	*NA*	*9.6E-06*	NA	Y					
Methyltestosterone											*NA*	*2.5E-06*	NA	B					
19-Nortestosterone											*NA*	*4.7E-06*	NA	B					
Test Case Chemical																			
D4											*NA*	*5.0E-06*	NA	I					

[A] Unless provided in the publication, Relative Potencies were derived by dividing an EC% value of the primary endogenous human estrogen, 17β-estradiol, by the equivalent EC% value of each chemical. $EC_{\%}$ values were either extracted from tables or interpolated from figures. Where necessary, concentrations were adjusted to better approximate equivalent EC% values. NA: Not Applicable

[B] Values derived from Table 4-1; ICCVAM, 2011. [BG1Luc: human ovarian cell line, ERα reporter]

[C] Values derived from Table 3; Jefferson et al., 2002. [BG1Luc: human ovarian cell line, ERα reporter]; molar values were calculated from the published data as shown in columns on right end of the table labeled Molar Conversion Calculations.

[D] Values derived from Table 2; Dornstauder et al., 2001. [YES: Yeast Estrogen Screen, human ERα reporter]

[E] Values derived from Table 3; Beck et al., 2003. [YES: Yeast Estrogen Screen, human ERα reporter]

[F] Values derived from Table 2; Pfitscher et al., 2008. [YES: Yeast Estrogen Screen, human ERα reporter]

[G] Values derived from Figure 1; Coldham et al., 1997. [YES: Yeast Estrogen Screen, human ERα reporter]

[H] Values derived from Table 1; Breinholt & Larsen, 1998. [YES: Yeast Estrogen Screen, human ERα reporter]

[I] Values taken from Figure 4; Quinn et al., 2007. [MCF-7: human breast adenocarcinoma cell line, ERα reporter]

[J] Values from Table 3; Procházková et al., 2017. [HeLa-9903; human cervical carcinoma cell line, ERα reporter]

[K] Values from Table 2; Gutendorf & Westendorf, 2001. [K-1: MVLN: Stably transfected MCF-7 cell line, ERα reporter / K-2: HGELN: stably transfected HeLa cells (see [J]), ERα reporter / K-3:MCF-7; human breast adenocarcinoma cell line, ERα reporter]

[L] Values from Figure 3a RP ratios; Mueller et al. 2004. [Ishikawa EREα: human endometrial adenocarcinoma cell line, ERα reporter]

[M] Values from Table 1, Ruotolo et al. 2013. [YES: Yeast Estrogen Screen, human ERα reporter]

[N] Values from Figure 1, Chrzan & Bradford, 2007. [N-1: MCF-7 (human breast adenocarcinoma cell line, ERα reporter / N-2: G-292: human osteogenic sarcoma cell line, ERα reporter]

[O] Values from Table 1, Chu et al. 2009. [YES-SRC-1: Yeast Estrogen Screen, SRC-1 human ERα reporter]

[P] Values from Table 2, Escande et al., 2006. [HELN: stably transfected HeLa cells (see J), ERα reporter]

[Q] Values from Table 2; De Angelis et al., 2005. [HEC-1: human endometrial adenocarcinoma cell line, ERα reporter]

[R] Values from Figures 2 & 3; Djiogue et al., 2014. [R-1: MVLN: Stably transfected MCF-7 human breast adenocarcinoma cell line, ERα reporter / R-2: U2OS: human osteosarcoma cell line, ERα reporter]

[S] Values from Table 1; Takeuchi et al., 2009. [CHO: Chinese Hamster Ovary cell line, ERα reporter]

[T] Values from Table 1; Islam et al., 2015. [U2OS: human osteosarcoma cell line, ERα reporter]

[U] Values from Table 2; Harris et al., 2005. [MCF-7; human breast adenocarcinoma cell line, ERα reporter]

[V] Values from Pianjing et al., 2011. [TD47-KBLuc: human breast cancer line, ERα reporter]

[W] Values from Zierau et al., 2002. [W-1: YES: Yeast Estrogen Screen, ERα reporter / W-2: MVLN: Stably transfected MCF-7 cell line, ERα reporter]

[X] Values from Zierau et al., 2004. [X-1: YES: Yeast Estrogen Screen, ERα reporter / X-2: MVLN: Stably transfected MCF-7 cell line, ERα reporter]

[Y] Values from Table 1 (PC_{10}, PC_{50}), Yamasaki et al., 2002b. [HeLa229 cell line, ERα reporter]
